# Magnetic resonance imaging of the coronary arteries

**Published:** 2007

**Authors:** SC Gerretsen, ME Kooi, JMA van Engelshoven, T Leiner, S Schalla, T Delhaas, G Snoep

**Affiliations:** Department of Radiology, Maastricht University Hospital, Maastricht, The Netherlands Cardiovascular Research Institute Maastricht (CARIM), University of Maastricht, Maastricht, The Netherlands; Department of Radiology, Maastricht University Hospital, Maastricht, The Netherlands Cardiovascular Research Institute Maastricht (CARIM), University of Maastricht, Maastricht, The Netherlands; Department of Radiology, Maastricht University Hospital, Maastricht, The Netherlands Cardiovascular Research Institute Maastricht (CARIM), University of Maastricht, Maastricht, The Netherlands; Department of Radiology, Maastricht University Hospital, Maastricht, The Netherlands Cardiovascular Research Institute Maastricht (CARIM), University of Maastricht, Maastricht, The Netherlands; Cardiovascular Research Institute Maastricht (CARIM), University of Maastricht, Maastricht, The Netherlands; Department of Cardiology, Maastricht University Hospital, Maastricht, The Netherlands; Cardiovascular Research Institute Maastricht (CARIM), University of Maastricht, Maastricht, The Netherlands; Department of Paediatrics, Maastricht University Hospital, Maastricht, The Netherlands; Department of Radiology, Maastricht University Hospital, Maastricht, The Netherlands

## Abstract

Despite progress in prevention and early diagnosis, coronary artery disease (CAD) remains one of the leading causes of mortality in the world. For many years, invasive X-ray coronary angiography has been the method of choice for the diagnosis of significant CAD. However, up to 40% of patients referred for elective X-ray coronary angiography have no clinically significant stenoses. These patients still remain subjected to the potential risks of X-ray angiography. As an alternative, magnetic resonance imaging (MRI) is currently one of the most promising techniques for noninvasive imaging of the coronary arteries. O ver the past two decades, many technical developments have been implemented that have led to major improvements in coronary MRI. Nowadays, both anatomical and functional information can be obtained with high temporal and spatial resolution and good image quality. In this review we will discuss the technical foundations and current status of clinical coronary MRI, and some potential future applications.

## Summary

Despite progress in prevention and early diagnosis, coronary artery disease (CAD) remains the leading cause of mortality in the world, accounting for 13% of all deaths worldwide.^1^,^2^ For many years, invasive X-ray coronary angiography has been the method of choice for the diagnosis of significant CAD (defined as more than 50% stenosis of the coronary artery lumen). Although several non-invasive tests are available to help discriminate between patients with and without significant angiographic disease, studies demonstrated that up to 40% of patients referred for elective X-ray coronary angiography have no clinically significant stenoses.^3^,^4^ Despite the absence of significant narrowing, these patients remain subjected to the potential risks of X-ray angiography. Therefore, development of a clinically useful non-invasive technique is desirable.

Of the non-invasive techniques, multi-detector row computed tomography (MDCT) and magnetic resonance imaging (MRI) are currently the most promising for non-invasive imaging of the coronary arteries. MDCT is a rapidly evolving technique with the main advantage of isotropic high spatial resolution. MRI, however, is the established standard technique for studying cardiac anatomical structure and function and does not involve exposure to ionising radiation. Furthermore, MRI contrast agents are much safer than the iodinated contrast agents, as evidenced by the much lower rate of allergic reactions and the absence of clinically detectable nephrotoxicity.^5^

This review will focus on the technical foundations and the current status of clinical coronary MRI. Since the coronary arteries were first depicted with MRI in the mid 1980s,^6^,^7^ many technical developments have been implemented that have led to major improvements in image quality. Nowadays it is possible to routinely visualise the proximal and middle parts of the coronary arteries and their branches with high accuracy. In addition, development of vessel wall imaging techniques is promising for detection of wall abnormalities in the absence of significant CAD.

## Technical aspects of coronary magnetic resonance imaging

A thorough understanding of the relative merits and shortcomings of coronary MRI demands a basic knowledge of equipment and technical principles, which will be discussed below. Currently, coronary MRI demands a compromise between spatial resolution, scan duration and vessel-to-background contrast.

## Equipment considerations

State-of-the-art coronary MRI is performed on 1.5 tesla (T) MR systems using dedicated cardiac phased-array radio-frequency coils applied to the chest wall. Technological advances in MR system architecture now enable accelerated data acquisition by simultaneously using 16- or 32-receiver channels.^8^

Appropriate cardiac receiver coils are required to meet the high in-plane spatial resolution requirements for coronary MRI while maintaining a sufficient signal-to-noise ratio (SNR) in comparison with use of the standard system built-in body coil. The use of these coils should be standard for all coronary MRI examinations.^9^ Because the SNR decreases with the distance from the surface receiver coil, cardiac specific coils have been optimised for the size of the heart and the distance of the heart from the chest wall. The right, left main and left anterior descending (LAD) coronary arteries are located relatively close to the anterior chest wall and can therefore be visualised with good image quality. However, the more distal parts of the circumflex artery are more difficult to depict.

With phased-array coils, parallel imaging techniques such as sensitivity encoding (SENSE),^10^ simultaneous acquisition of spatial harmonics (SMASH),^11^ and generalised autocalibrating, partially parallel acquisitions (GRAPPA)^12^ can be used to accelerate image acquisition by using the locally differing sensitivities of the separate receiver-coil elements. The acceleration factor is dependent on the coil type, and is typically two- to three-fold. With the newest generation of 16- and 32-receiver channel systems, even higher factors can be reached.^8^,^13^ However, a trade-off must be made between image quality and scan duration since the use of high-acceleration factors will lead to decreased SNR and contrast-to-noise ratio (CNR).^10^,^14^

**Fig. 1.**
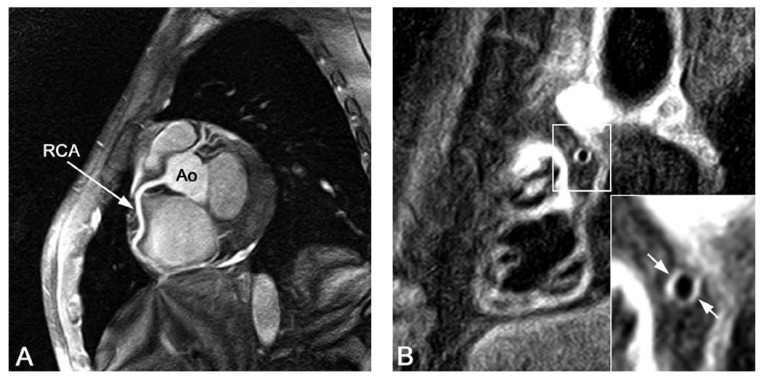
Three-tesla images of the right coronary artery (RCA). (A) Three-dimensional gradient echo sequence of the RCA using a T2 pre-pulse and fat saturation. (B) Dual inversion recovery (DIR) and fat-saturated gradient echo sequence of the RCA vessel wall. Both images show a high signal-to-noise ratio (SNR). The coronary vessel wall is clearly visible in B (arrows). Ao: aorta. (Courtesy M Stuber, Johns Hopkins University, Baltimore, MD, USA.)

In recent years, 3.0 T high-field MRI systems have become more widely available and are starting to be used for coronary imaging. The potential doubling of the SNR at 3.0 T could lead to further progress in cardiac applications, including coronary MRI. However, increased susceptibility effects and specific absorption rate (SAR) limitations due to altered penetration of radio-frequency (RF) pulses are potentially disadvantageous.^15^ Nevertheless, first results of cardiac and coronary MRI at 3 T are promising (Fig. 1) and show increased SNR and CNR compared to the results at 1.5 T.^16^-^18^

However, the theoretically predicted two-fold gain in SNR has not yet been achieved and current techniques do not result in significantly improved image quality and diagnostic accuracy compared with the quality and accuracy at 1.5 T.^19^ On the other hand, Huber *et al.* found the added SNR at 3 T to be sufficient to use parallel imaging with a reduction factor of two. This resulted in a 50% reduction in scan duration, with largely preserved image quality despite inevitable SNR loss relative to conventional full acquisition at 3 T.^20^

## Spatial resolution requirements

Coronary MRI data acquisition is technically challenging because of the tortuosity and small calibre (on average 1.5−5.5 mm^21^) of the coronary arteries. In this context, it is important to realise that accurate assessment of the degree of stenosis demands at least three pixels across the normal vascular lumen, as has been demonstrated by Hoogeveen *et al.*^22^ This constraint imposes a sub-millimetre in-plane resolution on coronary artery MRI protocols, especially in protocols used for stenosis detection.

The use of modern MR systems in combination with dedicated surface coils and optimised pulse sequences readily enables sub-millimetre in-plane resolution with current best pixel sizes of around 0.7 × 0.7 mm and slice thickness in the order of 1 mm. Despite satisfying the resolution requirement, the spatial resolution of coronary MRI is currently lower compared to the resolution of X-ray angiography (in the order of 0.2−0.3 mm), and MDCT (pixel sizes of around 0.5 mm). A drawback of coronary MRI is that higher-resolution images increase acquisition time and lead to lower SNR when all other parameters are kept constant.

## Motion compensation

Because the MR data acquisition process is inherently motion sensitive, techniques must be applied to compensate for cardiac and respiratory motion. During the cardiac cycle, in-plane coronary artery displacement can be up to 5 mm.^23^,^24^ The right coronary artery is more motion sensitive than the left coronary system. The data points needed to reconstruct an image of the heart and coronary arteries are generally obtained over multiple consecutive cardiac cycles. This strategy is also known as ‘segmented’ *k*-space sampling and demands accurate four-lead vector ECG registration^25^ and a regular cardiac rhythm.

Studies by Hofman *et al.* and Wang *et al.* have demonstrated that coronary artery motion is minimal during mid-diastolic diastasis, the cardiac rest period,^24^,^26^ and that best image quality is obtained when the acquisition window is optimised by using a subject-specific trigger delay^27^ after detection of the R-wave. The length of both the end-systolic and mid-diastolic rest periods depends on heart rate and can be individually determined from high temporal resolution ciné images. To obtain good image quality, an individually tailored acquisition window, preferably less than 120 ms, is advised.^24^,^28^,^29^

An additional important source of motion artefacts is beat-to-beat variations in heart rate and premature heartbeats. Leiner *et al.* have demonstrated improved coronary MR image quality with the use of combined respiratory gating and arrhythmia rejection.^30^ The possibility of exactly tailoring acquisition duration and correcting for variations in heart rate is a major strength of MR imaging. In contrast, MDCT acquisition times are generally not individually tailored and almost always exceed the length of mid-diastolic diastasis. This has led to preventative use of beta-blockers to lower heart rate during CT imaging, a strategy not routinely used in MR imaging.

In addition to taking into account cardiac contraction, respiratory motion has to be compensated for. At present, two techniques are used for respiratory motion compensation: holding the breath, and navigator gating. Holding the breath is highly dependent on patient cooperation. In addition, patients with pulmonary disease or heart failure are often not able to hold their breath long enough. Furthermore, slow cranial shifting of the diaphragm (drift) during breath hold can still cause motion of the heart and the coronary arteries during acquisition.^31^

The navigator gating technique, on the other hand, uses a two-dimensional pencil beam that is placed on an interface that reflects respiratory motion, eg the lung−liver or lung−myocardium interface.^32^ The navigator monitors the motion of this interface during free breathing. Data are accepted only when the selected interface falls within a user-defined window positioned around the end-expiratory level of the interface. With this technique, less patient cooperation is required. However, diaphragmatic drift can also occur during free breathing. Therefore, drift correction of the navigator window during the scan is essential to maintain sufficient efficiency and reasonable scan duration.

In terms of image quality and diagnostic accuracy, the free-breathing navigator technique is superior to breath-hold coronary MRI.^33^,^34^ Because of longer scan duration (not limited by breath hold), better SNR or spatial resolution can be achieved.

## Pulse sequences and vessel-to-background contrast

The optimal coronary MRI sequence has not yet been established. Essential elements are cardiac gating, respiratory motion suppression and pre-pulses to increase the signal of coronary arterial blood in relation to surrounding tissue (ie, increased CNR). Furthermore, SNR should be optimised while at the same time keeping scan duration within acceptable limits.

Both bright-blood and black-blood techniques have been evaluated in two-dimensional (2-D) and three-dimensional (3-D) pulse sequences. Bright-blood sequences tend to overestimate atherosclerotic lesions because of artificial darkening caused by focal turbulent flow.^35^ The vessel luminal diameter may therefore be underestimated in comparison with conventional X-ray angiography. On the other hand, the signal intensity of thrombus, vessel wall and various components of plaque may appear high on bright-blood coronary MR angiography (MRA), thereby obscuring focal stenoses.

Despite these drawbacks, the majority of sequences for coronary artery lumen imaging are bright-blood approaches with 2-D or 3-D gradient echo sequences with Cartesian segmented *k*-space sampling. Two-dimensional breath-hold coronary MRA has been shown to be a promising and valuable method for assessment of the native coronary arteries. With 3-D methods, scan duration is longer but these methods also have inherent advantages, including minimising bulk cardiac motion, superior SNR, the acquisition of thin contiguous sections and the ability to post-process and reformat the data set.^36^ Recently, bright-blood balanced steady-state free-precession (bSSFP) sequences have gained considerable interest. SSFP imaging is a very promising technique for coronary MRA MRI at 1.5 T with high SNR and CNR.^37^ Nevertheless, this sequence is more sensitive to magnetic field inhomogeneities. The increased main field inhomogeneity and B1 field variations at 3 T can potentially be problematic for SSFP imaging.^16^

By contrast, black-blood coronary imaging is performed using spin echo sequences. With this technique, there is a potential for enhancing CNR in comparison with gradient echo approaches. In addition, black-blood sequences appear to be particularly advantageous for patients with implants such as vascular clips or sternal wires because spin echo techniques are less sensitive to the susceptibility artefacts from metallic implants.^38^,^39^

**Fig. 2.**
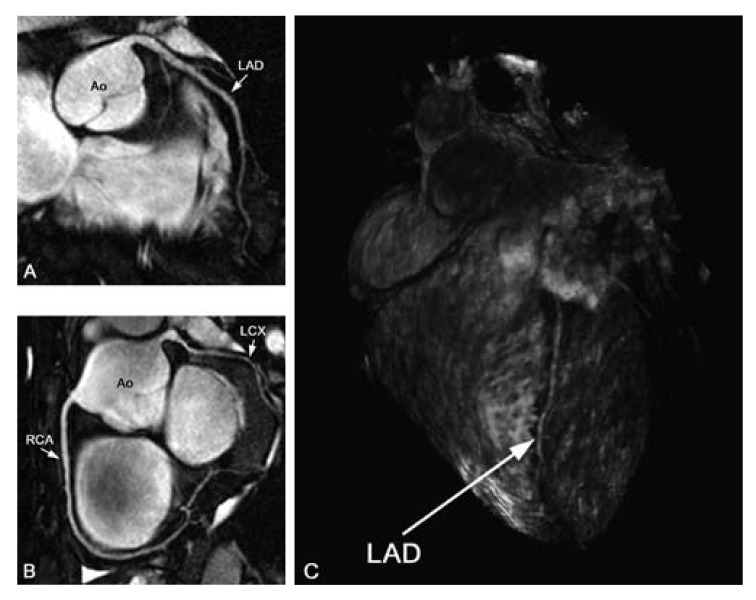
(A) Targeted images of the left anterior descending artery (LAD), (B) right coronary artery (RCA) and (C) whole-heart scan in healthy volunteers. With the targeted approach, high-resolution images of the coronary arteries and some branches can be obtained in a relatively short scan time. By using a whole-heart approach, all coronary arteries are imaged in one scan, and the data set allows for post-processing and display of anatomy similar to multi-detector computed tomography. LCX: left circumflex artery, Ao: aorta.

The coronary arteries can be imaged using either a double-oblique, targeted approach or a whole-heart scan. With the former technique, a 3-D slab is acquired in a user-defined orientation around the coronary arteries. The vessel of interest can be covered with high spatial resolution, and scan duration is shorter compared to whole-heart MRI. Although extensive parts of the coronary arteries can be depicted with this technique,^36^ multiple scans are required since not all coronary arteries can be covered with one scan (Fig. 2A, B). The whole-heart technique, first described by Weber *et al.*,^40^ is a magnetisation prepared bSSFP sequence using an acquisition volume covering the whole heart. The major advantages of the technique are that positioning of the imaging volume is relatively simple, it facilitates high-quality coronary MRA of the complete coronary artery tree in a single measurement and it allows post-processing and display of data sets similar to MDCT (Fig. 2C). A drawback at present is the relatively long acquisition duration, although this will change with more widespread use of parallel imaging techniques.

An important aspect of coronary imaging is the contrast between coronary arteries and surrounding epicardial fat and myocardium. In comparison with MDCT, exogenous contrast media are not necessary to achieve coronary artery enhancement. Several techniques have been developed to increase CNR between the blood and myocardium. Perivascular fat can be suppressed by applying fat saturation^41^ or spectral pre-saturation with inversion recovery (SPIR) pre-pulses. Suppression of the myocardium can be achieved by the use of a T2-preparatory pre-pulse, a technique in which a dedicated pre-pulse is used to achieve a decreased signal from the myocardium while maintaining the signal from the blood, leading to an improved CNR between blood and myocardium and better vessel definition.^28^,^42^

The role of exogenous contrast agents remains to be established, as the use of contrast medium can actually reduce vessel-to-background contrast. For instance, commonly used extracellular agents extravasate into the myocardium and perivascular fat, thereby decreasing the contrast between the coronaries and the myocardium. The use of intravascular contrast agents could be an alternative since these agents exhibit prolonged intravascular retention and have a longer plasma half-life and shorter T1, resulting in higher signal intensity. This allows imaging over a longer period of time so navigator techniques can be used to obtain images of high quality with high blood/muscle contrast and better vessel delineation^43^,^44^ In addition, contrast agents may become an important adjunct to coronary imaging at 3 T as these agents ameliorate some of the imaging artefacts encountered at higher field strengths.^45^

## Clinical indications for coronary MRI

## Detection of anomalous coronary arteries

Although congenital coronary artery anomalies are relatively uncommon – the estimated incidence is about 1% in the general population – they can be the cause of severe myocardial ischaemia, infarction or sudden cardiac death.^46^ These adverse events commonly occur during or immediately following intense exercise and are thought to be related to compression or proximal angulation, which obstructs blood flow within the anomalous vessels, leading to distal ischaemia, ventricular tachycardia and ventricular fibrillation.^47^ Therefore, it is vital that the precise anatomical arrangement is identified to enable the appropriate management plan to be followed.^48^

**Fig. 3.**
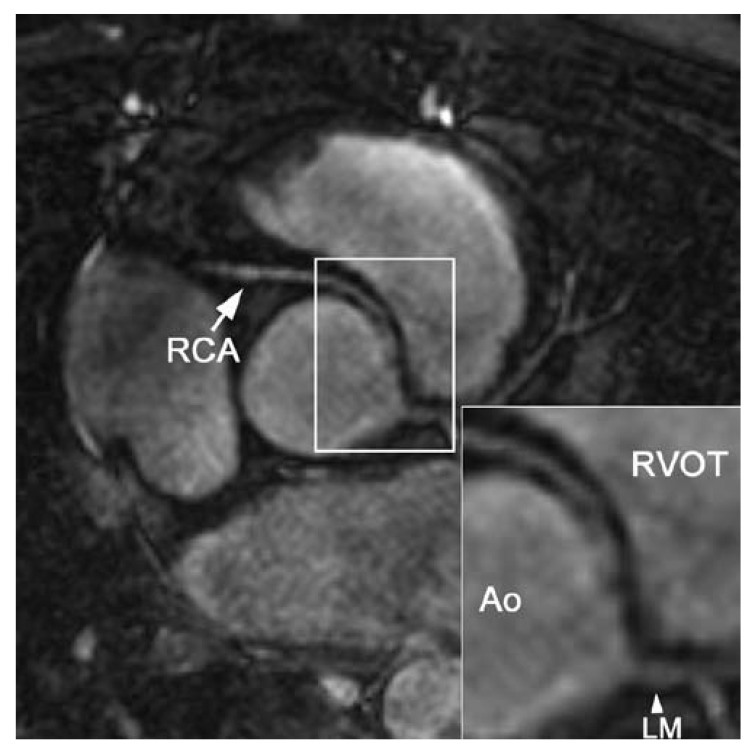
Three-dimensional balanced steady-state free precession (bSSFP) coronary MRI at 1.5 T in a 51-year-old female with aberrant right coronary artery (RCA). The RCA originates from the left coronary artery sinus and traverses between the aorta (Ao) and the right ventricular outflow tract (RVOT). LM indicates the left main stem.

Projection X-ray angiography used to be the imaging test of choice for the diagnosis and characterisation of these anomalies. However, detection of anomalies may be difficult and the exact anatomical course can be difficult to determine. One of the main advantages of using MRI instead of X-ray angiography is the visualisation of the coronary arteries in relation to other mediastinal structures such as the right ventricular outflow tract. Three-dimensional coronary MRI is well suited for the depiction of anomalous coronary artery origins (Fig. 3). Several studies have demonstrated that MR angiography is highly accurate in detecting anomalous coronary arteries and delineating the proximal course.^48^-^50^ Coronary MRI is now the method of choice in young patients in whom coronary artery anomaly is suspected or needs to be further clarified, or if the patient has another cardiac anomaly associated with coronary anomalies.^51^

**Fig. 4.**
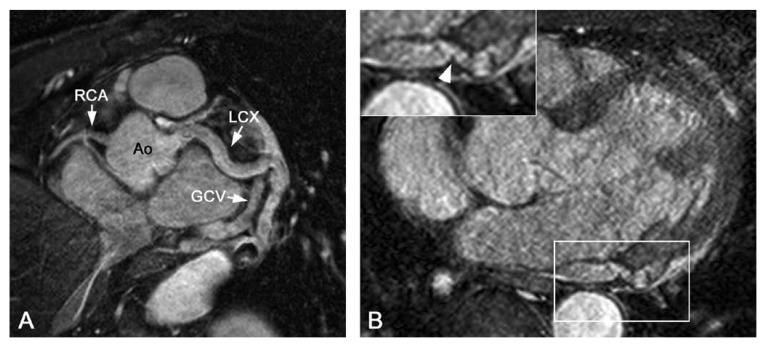
(A) Targeted Cartesian balanced steady-state free precession (bSSFP) and (B) whole-heart source image in a patient with a coronary arteriovenous fistula. Note the enlarged LCX compared to the left anterior descending artery (LAD) and right coronary artery (RCA). There is a connection (arrowhead) between the left circumflex artery (LCX) and the coronary sinus. GCV: great cardiac vein, Ao: aorta.

A coronary artery fistula is the most important haemodynamically significant coronary artery anomaly. It is characterised by an abnormal communication between a coronary artery and a cardiac chamber, major vessel or other vascular structure. Surgical repair of the fistula is recommended for symptomatic patients and for asymptomatic patients at risk for future complications (steal, aneurysms, large shunts).^52^ As a non-invasive technique, MRI is very suitable for detection and follow-up of these fistulae (Fig. 4).

## Kawasaki disease and follow-up of coronary artery aneurysms

**Fig. 5.**
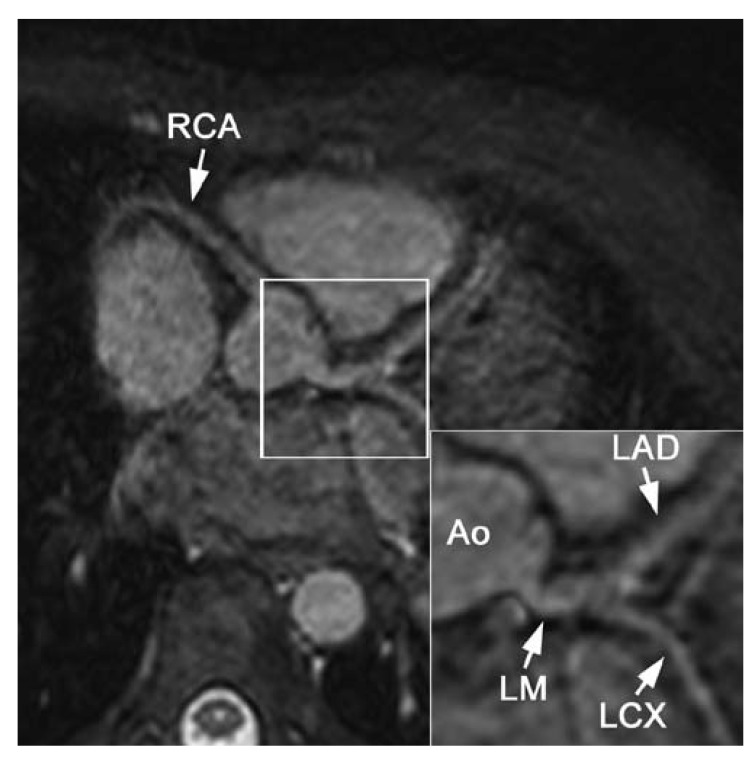
Post-processed whole-heart scan of a 12-year-old boy diagnosed with Kawasaki disease. Dilatation of the left and right coronary artery can be seen. Note the kinking and aneurysmatic distal part (diameter 4.4 mm) of the left main stem (LM) in comparison with the diameter of the ostium (diameter 2.8 mm). Ao: aorta, LAD: left anterior descending artery, LCX: left circumflex artery, RCA: right coronary artery.

Kawasaki disease, an acute vasculitis of unknown origin, is the leading cause of acquired coronary artery disease in children in developing countries and is now reported as a potential risk factor for adult ischaemic heart disease and sudden death in early adulthood.^53^ There is a 25% chance of serious cardiovascular damage if treatment is not initiated early in the course of the disease.^54^ Coronary damage, including dilatation, aneurysms (defined as coronary diameter > 4 mm) and giant aneurysms (coronary diameter > 8 mm) develop in up to 5% of timely treated patients. In addition to aneurysm development in infants and children, this syndrome may eventually lead to thrombotic occlusion, premature atherosclerosis and progression to ischaemic heart disease.^53^ Serial evaluation of coronary aneurysms is important, and regression of these aneurysms has been reported in approximately 50% of the patients.^55^ In young children, transthoracic echocardiography is usually adequate for detecting and following these aneurysms, but this technique often becomes inadequate as children grow. An alternative method for follow up is MRI, which is considered equivalent to coronary angiography^56^,^57^ (Fig. 5).

## Detection of stenoses in native coronary arteries

**Fig. 6.**
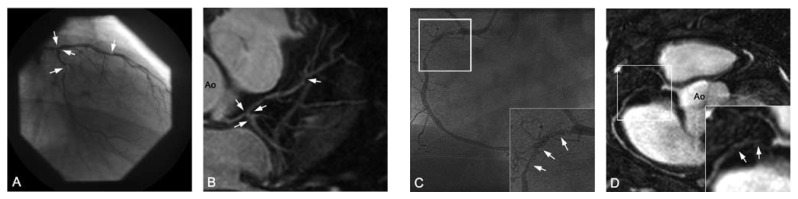
Correlation between coronary X-ray angiography and MRI. X-ray angiography (A) and a targeted Cartesian balanced steady-state free precession (bSSFP) MR sequence (B) in a 62-year-old patient with left coronary artery disease. X-ray angiography (C) and radial bSSFP images (D) in a different 53-year-old patient with stable angina. A diffuse, long and severe stenosis of the proximal right coronary artery (RCA) can be seen with both techniques. In both cases, there is a good correlation between angiography and MRI (arrows).

The most important potential clinical application of coronary MRI is detection of stenoses in native coronary arteries. For this purpose, bSSFP bright-blood techniques are mostly used where areas of focal stenosis produce signal voids of varying severity related to the angiographic degree of stenosis (Fig. 6). However, gradient echo coronary MRI may sometimes overestimate the degree of stenosis as blood flow alterations in stenotic segments can cause signal loss in the segment distal to the lesion.^58^ Similarly, the presence of heavy calcification in the coronary vessel wall may lead to signal voids that artificially suggest the presence of significant stenoses. On the other hand, adequate collateral blood flow is readily detected as it results in signal in the lumen distal to an occlusion.

A meta-analysis by Danias *et al*. summarising the diagnostic performance of 39 studies of coronary MRA found the technique to have moderately high sensitivity for detecting significant proximal stenoses and to be of value for exclusion of significant multi-vessel CAD in subjects with suspected CAD considered for diagnostic catheterisation (Table 1). The study found coronary MRA to have high diagnostic performance in all vessels except the left circumflex coronary artery, and it was particularly useful to rule out the presence of significant CAD to a probability below 5%, especially in individuals with modest suspicion for CAD (pre-test probability for CAD below 20%). However, this meta-analysis showed large heterogeneity of the results, indicating that the performance of coronary MRA is, at present, probably still centre-dependent.^59^

**Table 1:** Diagnostic Accuracy O F Coronary MRA Compared To Conventional X-Ray Angiography

*Analysis*	*Weighted sensitivity RE (%) (95% CI)*	*Weighted specificity RE (%) (95% CI)*
Segment level	73 (69−77)*	86 (80−90)*
Left main^†^	69 (56−79)	91 (84−95)*
LAD	79 (73−84)*	81 (71−88)*
LCx	61 (52−69)	85 (78−90)*
RCA	71 (64−78)*	84 (77−88)*
Subject level	88 (82−92)	56 (43−68)*
Vessel level	75 (68−80)*	85 (78−90)*

*Statistically significant (*p* < 0.10) between-study heterogeneity. ^†^Four studies evaluated the left main together with the proximal segment of the LAD. These data were included in the LAD analysis. CI = confidence interval; LAD = left anterior descending; LCx = left circumflex; RCA = right coronary artery; RE = random effects. [modified from: Danias et al. *J Am Coll Cardiol* 2004; **44**(9)]

Of note, the largest multi-centre coronary MRA study to date reported on 109 patients scheduled for elective X-ray angiography because of suspected CAD. This study found high sensitivity, modest specificity and high negative predictive value and overall accuracy of coronary MRA for the identification of coronary disease, especially in subjects with left main coronary artery disease or three-vessel disease.^3^

**Fig. 7.**
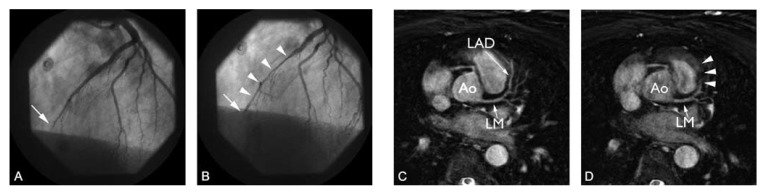
Coronary angiogram (A, B) and targeted Cartesian balanced steady-state free precession (bSSFP) MRI with mid-diastolic (C) and systolic imaging (D) in a 52-year-old man with acute anterior wall myocardial infarction. Coronary angiography revealed single-vessel disease of the left anterior descending artery (LAD). In A, distal occlusion of the LAD can be seen (arrow). B, systolic squeezing of the intramyocardial mid-LAD (arrowheads). C, the proximal and mid-segment of the LAD can be seen during diastole, however, this segment disappears during systole (D, arrowheads). Ao: aorta, LM: left main stem.

An infrequent cause of chest pain is myocardial bridging, a condition in which part of a coronary artery is situated in the myocardium and compressed during systole. Long tunnelled segments of coronary arteries, more severe systolic diameter narrowing of the tunnelled segment and tachycardia may result in myocardial ischaemia.^52^ MRI using systolic and diastolic acquisition windows can be used to non-invasively detect and follow patients with myocardial bridging^60^ (Fig. 7).

## Assessment of coronary artery bypass grafts (CABG)

Occlusion or stenosis of grafts can occur after coronary artery bypass grafting and this incidence increases over time.^61^ Because of their fixed position and large lumen size, bypass grafts are relatively easy to image despite possible artefacts due to the presence of sternal wires or metal clips. Langerak *et al.* have demonstrated that MRI can be used to determine graft patency and to assess the presence of vein graft disease with a fair diagnostic accuracy. Sensitivity and specificity for identifying graft occlusion and stenosis ranged from 65 to 83% and 80 to 100%, respectively.^62^

## Intracoronary stents

Nowadays, intracoronary stents are routinely used as adjunct to percutaneous coronary artery revascularisations for CAD. Early MRI can be safely performed as early as one to three days after stent implantation.^63^-^66^ Depending on the material of the stent, local susceptibility effects lead to signal voids and artefacts. The size of the artefact is influenced by the type of MR sequence (larger with gradient echo sequences), imaging parameters and stent material (less with titanium, larger with stainless steel).^67^,^68^ At present, these artefacts prevent the direct visualisation of in-stent restenosis. Recently, a new MR-lucent stent was developed by Spuentrup *et al.* In an animal model, this stent allowed for completely artefact-free coronary MRA and vessel wall imaging.^68^,^69^

## Flow and perfusion measurements

The functional significance of a stenosis cannot only be determined by assessment of the anatomical severity. In a substantial number of cases, functional information is therefore obtained by measuring coronary blood flow and flow reserve. The latter parameter is the ratio of maximal hyperaemic coronary flow to baseline coronary flow.^70^ Assessment of the functional significance of a stenosis is particularly important in lesions with intermediate severity because the interpretation of such lesions significantly influences therapeutic decisions in patients with coronary artery disease.^71^

Fast phase-contrast ciné MRI is an application that can provide non-invasive assessment of blood flow and flow reserve in human coronary arteries. The technique has recently been used for detection of flow in native coronary arteries before and after interventions, and in bypass grafts for detection of graft stenosis.^70^,^72^ Additionally, MRI of coronary anatomy and flow can be combined with wall motion and stress perfusion studies.^73^-^76^

## Future developments: vessel wall and plaque imaging

Although luminography is the established method for detection of atherosclerotic lesions, it is well known to underestimate early atherosclerosis, since atherosclerotic plaques can be present without visible luminal narrowing. This phenomenon was first described by Glagov *et al.* and is also known as ‘positive remodelling’. 77,78 Falk *et al.* and Schoenhagen *et al.* found that patients with only mild-to-moderate luminal narrowing actually have a higher risk of a future acute coronary syndrome when compared to patients with more significant luminal narrowing.^79^,^80^ This is especially the case when plaques have a large lipid-rich necrotic core and a thin fibrous cap.

Since these publications, MR vessel wall imaging has become a topic of considerable interest. Because of the superior ability of MRI to differentiate different soft tissues, it is uniquely suited for non-invasive serial imaging of the arterial vessel wall, which might be useful for monitoring progression or regression of atherosclerotic plaque during treatment of atherosclerosis with statins,^81^ even in the absence of significant luminal narrowing.^82^ Additionally, MRI can potentially be used for risk stratification, eg, identification of those plaques with increased risk of causing ischaemic complications. MR imaging of the vessel wall has been performed in the aorta^83^ and the carotid arteries.^84^,^85^ In these vessels, detection and characterisation of plaque components is feasible. Fayad *et al.* and Botnar *et al.*^29^ were the first to succeed in using MRI to directly visualise coronary vessel wall disease. However, MRI of the coronary vessel wall is still considered to be an experimental technique.

The coronary vessel wall can be visualised with high spatial resolution in cross-sectional or longitudinal fashion. With MRI, increased coronary vessel wall thickness and wall area could be detected in patients with angiographically proven CAD compared to healthy volunteers.^29^,^86^ In addition, Kim *et al.* found that positive remodelling in patients with non-significant coronary artery disease could be detected.^87^ Furthermore, Desai *et al.* demonstrated that assessment of coronary vessel wall thickness from MR images is highly reproducible.^88^ In contrast to larger arteries such as the carotids, however, in vivo detection of plaque components in human coronaries is currently limited by scan duration, SNR and spatial resolution.

Coronary vessel wall thickness is only 0.5−0.75 mm in healthy young subjects. In the diseased vessel wall, thickness increases to over 1 mm. With current pixel sizes of about 0.7 × 0.7 mm, only one or two pixels usually cover the vessel wall. For accurate plaque characterisation however, at least one pixel in each vessel wall layer should be present.^89^ In addition, motion compensation strategies for coronary vessel wall imaging are more stringent than for coronary MRA.^27^ The feasibility of coronary vessel wall imaging at 3 T has recently been demonstrated. With more experience with higher field systems, further improvements in scan duration, resolution and image quality can be expected.^90^,^91^

**Fig. 8.**
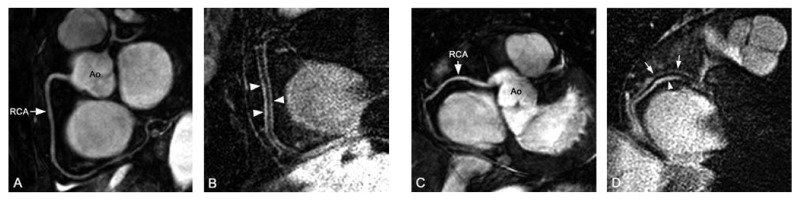
Radial balanced steady-state free precession (bSSFP) (A) and vessel wall scan (B) of the right coronary artery (RCA) in a 62-year-old healthy female. The proximal and middle parts of the RCA were free of atherosclerotic disease. The vessel wall (arrowheads) is well delineated, thin and has a uniform signal intensity. BSSFP (C) and vessel wall scan (D) in a 59-year-old female without history of coronary artery disease. In D, note the thickening and bright signal intensity of the posterior wall of the RCA (arrowhead) compared to the anterior wall (arrows).

MR coronary vessel wall imaging can be performed with several techniques. To distinguish the vessel wall from the lumen, the use of dual inversion pre-pulses for suppression of blood signal is essential. Two-dimensional techniques perpendicular to the vessel axis have been used, but with these techniques there is limited coverage of the artery of interest. *In vivo* multi-sequence imaging, as was done previously in, for instance, the carotid arteries,^92^,^93^ is very time consuming in the coronary arteries.^94^ By contrast, 3-D imaging offers the opportunity for more extensive longitudinal coverage of the coronaries, improved SNR and higher spatial resolution^95^ (Figs 1, 8)).

Another promising development in the field of (coronary) plaque imaging is the use of contrast agents targeted to thrombus or endothelial cell surface markers. For instance, a fibrin-binding MR contrast agent has successfully been used for the detection of fresh thrombus in a variety of animal models and humans.^96^-^99^ When conventional extracellular agents are used, a delayed enhancement-like phenomenon, similar to that observed in stunned myocardium, can be observed within the coronary vessel wall.^100^,^101^ The exact significance of this finding remains to be elucidated, however.

## Coronary MRI in relation to other techniques

There are many techniques, both non-invasive as well as invasive, that can be used to obtain information about the coronary artery lumen and vessel wall. Invasive techniques include X-ray coronary angiography, coronary intravascular ultrasonography (IVUS),^102^-^104^ optical coherence tomography (OCT),^105^-^108^ angioscopy, 109,110 intravascular MRI^111^ and thermography.^112^,^113^ Except for IVUS, these techniques are currently only used in pre-clinical studies, but they might become useful in detection of coronary (vulnerable) plaque.

The most promising non-invasive alternative to MRI is MDCT, which can be performed with reliable results in selected patient populations, especially with the latest generation of 64-detector row scanners which combine thin-slice collimation with short gantry rotation times.^114^ Currently, MDCT is used for the evaluation of patients with a low pre-test likelihood of a significant coronary stenosis, patients with recurrent angina, follow-up of patients with previous coronary artery bypass grafting or coronary stents when they are located in proximal branches, the evaluation of chronic total coronary occlusion before percutaneous recanalisation, detection of coronary artery anomalies, and for the quantification of coronary artery calcium.^115^ A pre-clinical application of coronary CT is plaque imaging. It has been reported that coronary CT angiography has the potential to detect coronary plaques, quantify their volumes and eventually characterise their composition.^116^,^117^ However, extensive calcification has a major influence on these results, preventing adequate assessment of plaque composition in combined calcified/non-calcified plaques.

It is clear from the previous discussions that there are still questions about the future roles of both CT and MRI for coronary artery imaging. The evaluation of CAD by MRI or CT uses various strategies: detection of coronary calcifications (CT), direct imaging of coronary artery stenoses (MRI or CT) and detection of reduced coronary perfusion reserve (stress ciné MRI and stress perfusion MRI).^118^ Both CT and MRI can be used for direct imaging of atherosclerotic lesions, measurement of atherosclerotic burden and possibly characterisation of plaque components.^119^ Appropriate selection of patients is important for the successful application of these emerging imaging modalities.

At present both MRI and MDCT have proven to be clinically useful in the assessment of individuals with low and intermediate pre-test probability of significant CAD. Patients with high pre-test probability are best served by CAG.^118^ For emergency patients presenting with atypical chest pain, MDCT has major advantages because it is fast and the coronary arteries can be visualised with high isotropic spatial resolution while at the same time the presence of pulmonary embolism and aortic dissection can be assessed. However, screening for coronary artery disease or the follow-up of patients is not advised since the use of iodinated contrast agents is not without risks and radiation dose is high, varying from 1.5−16.3 mSv for MDCT (compared to 3−5 mSv for conventional CAG).^120^,^121^

MRI is more favourable for screening and follow-up because of its lack of ionising radiation and the use of safer contrast agents, but it currently lacks spatial resolution. However, intrinsic contrast-resolution is higher for MRI compared to CT. This is advantageous in tissue characterisation. Another major advantage of MRI is the integration of anatomical coronary imaging in a more comprehensive cardiac examination in which cardiac morphology, global cardiac function, regional wall motion and the extent of myocardial infarction can be assessed.

## Conclusions

Coronary MRI is a reliable, non-invasive and patient-friendly technique that can be used in combination with perfusion and wall motion studies to assess the presence of coronary artery anomalies, for follow-up of patients with coronary artery aneurysms as a complication of Kawasaki disease, to rule out proximal coronary artery stenoses in patients with a low and intermediate pre-test likelihood of CAD, and to detect myocardial infarction. The development of MR coronary vessel wall imaging and contrast agents targeted to plaque components will allow for fundamental *in vivo* insight into plaque development. Additional benefits can be expected from the transition to higher field-strength systems and the implementation of parallel imaging techniques in combination with dedicated coils and blood-pool contrast agents.
